# Routine Magnetic Resonance Imaging at Term-Equivalent Age Detects Brain Injury in 25% of a Contemporary Cohort of Very Preterm Infants

**DOI:** 10.1371/journal.pone.0169442

**Published:** 2017-01-03

**Authors:** Vera Neubauer, Tanja Djurdjevic, Elke Griesmaier, Marlene Biermayr, Elke Ruth Gizewski, Ursula Kiechl-Kohlendorfer

**Affiliations:** 1 Department of Paediatrics II, Neonatology, Medical University of Innsbruck, Innsbruck, Austria; 2 Department of Neuroradiology, Medical University of Innsbruck, Innsbruck, Austria; 3 Neuroimaging Core Facility, Medical University of Innsbruck, Innsbruck, Austria; Medizinische Universitat Innsbruck Department fur Kinder- und Jugendheilkunde, AUSTRIA

## Abstract

**Introduction:**

In recent years, significant investigation has been undertaken by means of magnetic resonance imaging (MRI) in an attempt to identify preterm infants at risk for adverse outcome. The primary objective is to provide a comprehensive characterization of cerebral injury detected by conventional MRI at term-equivalent age in an unselected, consecutive, contemporary cohort of preterm infants born <32 gestational weeks. Secondly, this study aims to identify risk factors for the different injury types in this population.

**Methods:**

Data for all preterm infants born <32 gestational weeks and admitted to Innsbruck Medical University Hospital were prospectively collected (October 2010 to December 2015). Cerebral MRI was evaluated retrospectively using a validated scoring system that incorporates intraventricular haemorrhage (IVH), white matter disease (WMD) and cerebellar haemorrhage (CBH).

**Results:**

300 infants were included in the study. MRI showed 24.7% of all infants to have some form of brain injury. The most common injury type was IVH (16.0%). WMD and CBH were seen in 10.0% and 8.0%. The prevalence of common neonatal risk factors was greater within the group of infants with CBH. In particular indicators for respiratory disease were observed more often: longer ventilation duration, more frequent need for supplemental oxygen at day 28, higher rates of hydrocortisone treatment. Catecholamine treatment was the only neonatal risk factor that was overrepresented in infants with WMD

**Discussion:**

Cerebral MRI at term-equivalent age, as addition to cranial ultrasound, detected brain injury in 25% of preterm survivors. The diagnosis of IVH was already made by neonatal ultrasound in most cases. In contrast, only a minority of the CBH and none of the non-cystic WMD have been detected prior to MRI. Decreasing gestational age and neonatal complications involved with immaturity have been identified as risk factors for CBH, whereas WMD was found in relatively mature infants with circulatory disturbances.

## Introduction

In European countries 1.1% to 1.6% of live births are very preterm.[1] Depending on gestational age, up to 49% of preterm infants exhibit a psychomotor or mental delay at toddler age.[2] This delay does not even out during the following years, but particularly cognitive and behavioural deficits are still or first become evident at school age and beyond.[3, 4] Despite numerous investigations that aimed to early identify infants at risk, prediction of long-term outcomes for individual infants is still challenging.

The use of magnetic resonance imaging (MRI) has provided an additional means of depicting the wide spectrum of preterm brain injury. This is of major importance since a key paper by Woodward et al. found that abnormal findings on MRI at term-equivalent age are significantly better at predicting adverse outcome than are abnormalities that can be detected by ultrasound.[5] Since then, several MRI evaluation scales have been developed to quantify the severity of brain abnormality and predict neurodevelopmental outcome of preterm infants.[5–7] However, these scoring systems usually account for cerebral white and grey matter, but disregard cerebellar injury and may thereby underestimate the full extent of injury.[5–7] Furthermore, several parameters in these scorings are considerably subjective. We thus chose to employ a recently developed simple scoring system that incorporates all major injury types and differentiates between intraventricular haemorrhage (IVH), white matter disease (WMD, including non-cystic and cystic WMD) and cerebellar haemorrhage (CBH).[8] There is evidence of associations between MRI findings and neurodevelopmental outcomes, but due to the above-mentioned reservations reported relationships are quite variable.[5, 9, 10] Another limitation of these studies is the fact that MRI was performed in a research setting and not as part of routine care. Thus, data on the absolute frequency of brain injury in unselected preterm populations are scarce and the analysis of associations with long-term outcome is hindered.

As a first step to a thorough work-up of this topic the present study was designed to provide a comprehensive characterization of cerebral injury detected by routine MRI at term-equivalent age in an unselected, consecutive, contemporary cohort of preterm infants born at <32 gestational weeks. Secondly, this study aims to identify risk factors for the various injury types in this population.

## Methods

### Patients

This study was a retrospective analysis of prospectively collected data. All infants born at <32 gestational weeks at Innsbruck Medical University Hospital, offering the only neonatal intensive care unit in Tyrol (Austria), between October 2010 and December 2015 were enrolled. Of all 383 live-born infants twelve children died and 16 were excluded because of congenital anomalies. Thus, 355 infants were eligible for MRI at term-equivalent age. Of these children 24 (6.8%) infants were transferred out of Tyrol prior to term-equivalent age, two (0.5%) infants were too unstable and 23 (6.5%) of all parents did not consent to participate. Accordingly, 306 (86.2%) infants were scanned at term-equivalent age; for six (1.7%) infants it was not possible to obtain high-quality MR images. Thus, the final study population consisted of 300 (84.5%) infants.

Cerebral MRI at term-equivalent age is part of our routine follow-up program for all preterm infants born at <32 gestational weeks. All infants were scanned without sedation during postprandial sleep as described in a previous paper.[11] All caregivers gave written informed consent to the performance of the MRI.

The study was approved by the ethics committee of the Medical University of Innsbruck (study No. AN2013-0086 333/4.2).

### Patient characteristics

Neonatal data was collected during the hospital stay as described previously.[12] Cranial ultrasound examinations were routinely performed during the initial hospital stay. All images were evaluated regarding the diagnoses of IVH, WMD and CBH in daily interdisciplinary meetings (neonatology, paediatric radiology).

### Magnetic resonance image acquisition

All images were acquired with a 3.0 Tesla Siemens Magnetom Verio (Siemens, Erlangen, Germany) at the local Department of Neuroradiology. The MRI protocol included the following imaging sequences: axial T2-weighted TSE images covering the whole head (TE 99 ms, TR 4590 ms, FOV 15 x 11 cm, matrix: 147 x 256, slice thickness 3 mm, no gap); 3D MP-RAGE T1-weighted images covering the whole head (TE 4.54 ms, TR 1770 ms, TI 1000 ms, flip angle 9 degrees, FOV 20 x 15 cm, matrix 144 x 192, slice thickness: 1.0 mm, gap 0.5 mm).

From 2012 susceptibility weighted imaging (SWI) was included in the routine protocol (available for 181 of 300 infants (60.3%)): axial SWI images covering the whole head including brain and skull (TE 20 ms, TR 27 ms, FOV 20 x 15 cm, matrix 182 x 256, slice thickness 2.0 mm, gap 0.4 mm). The use of SWI did not increase the rate of detection of cerebellar or intraventricular haemorrhages as compared to infants in whom SWI was not employed.

### Magnetic resonance image evaluation

Cerebral injury was graded according to a scoring system previously published by Kidokoro et al.[8] Kidokoro’s current brain injury assessment covers three common injury patterns in preterm infants (IVH, WMD and CBH).[8] All injury types were graded as grade 1 to grade 4 according to the degree of severity. High-grade injury (grade 3 or 4) in any category was defined as severe injury.

IVH grade 1 was defined as the presence of hemosiderin deposits or post haemorrhagic cysts within the thalamo-caudal notches. IVH grade 2 IVH was defined as the presence of hemosiderin deposits outside the region of the thalamo-caudal notches along the ventricular wall without ventricular dilatation. IVH grade 3 was defined as ventricular dilatation >97^th^ percentile with evidence of previous ventricular haemorrhage. IVH grade 4 was defined as the presence of parenchymal haemorrhagic lesions or posthaemorrhagic cystic encephalomalacia.

WMD grades 1 and 2 were defined by the presence of small punctate lesions (≤3 mm in individual size) in periventricular white matter on either or both of the T1/T2-weighted images. WMD grade 2 was differentiated from grade 1 by the presence of lesions in bilateral corticospinal tracts or with ≥3 lesions per hemisphere. WMD grade 3 was defined as the presence of extensive lesions along the wall of lateral ventricles with high signal on T1-weighted images. WMD grade 4 was defined as the presence of cystic lesions in periventricular white matter.

CBH grade 1 consisted of unilateral small punctate lesions (≤3 mm in size). CBH grade 2 consisted of bilateral small punctate lesions (≤3 mm in size). CBH grade 3 consisted of an extensive unilateral lesion (>3 mm in size). And CBH grade 4 was defined as bilateral extensive lesions (>3 mm in size).

High-grade injury (grade 3 or 4) in any category was defined as severe injury.

All MR images were evaluated by two operators (V.N., T.D.) blinded to the clinical data. Consensus was reached upon discussion.

### Statistical analysis

Data analysis was performed using SPSS software, version 22.0 for Windows (IBM Corp., Armonk, NY, USA). Data distribution was tested using the Kolmogorow-Smirnov test. Depending on the distribution of data, Student’s T test or the Mann-Whitney U test was employed for comparison of two groups. Comparison of categorical data was made with the chi-square or Fisher’s exact test.

## Results

### Patient characteristics

The maternal and neonatal characteristics of the 300 study participants are shown in Table 1. MRI was performed at a mean gestational age of 40.6 ±0.7 weeks.

**Table 1:** Neonatal characteristics of the study participants (n = 300).

Variable	n (%), mean (SD), median (range)
Gestational age at birth (weeks)	29.4 ±2.0
Gestational age <28 weeks	60 (20.0%)
Birthweight (grams)	1260 ±386
Small for gestational age	22 (7.3%)
Birthweight <1000g	82 (27.3%)
Male sex	155 (52.7%)
Inborn	284 (94.7%)
Multiple birth	129 (43.0%)
Caesarean delivery	278 (92.7%)
5’ Apgar	8 (3–10)
5’ Apgar <5	5 (1.7%)
5’ Apgar <7	32 (10.7%)
Antenatal steroids	275 (91.7%)
Surfactant treatment	233 (77.7%)
Ventilation (hours)	6 (0–1128)
Ventilation >6 hours	135 (45.0%)
Nasal continuous airway pressure ventilation (days)	7 (0–72)
Need for supplemental oxygen at day 28	77 (25.7%)
Need for supplemental oxygen at 36 weeks	28 (9.3%)
Postnatal hydrocortisone treatment	46 (15.3%)
Caffeine duration (postmenstrual age, weeks)	34.3 (32.0–43.9)
Preterm premature rupture of membranes >24 hours	54 (18.0%)
Early-onset sepsis (culture proven)	15 (5.0%)
Late-onset sepsis (culture proven)	31 (10.3%)
Patent ductus arteriosus	101 (33.7%)
Surgical ligation of a patent ductus arteriosus	11 (3.7%)
Catecholamine treatment	25 (8.3%)
Retinopathy of prematurity grade 3 or 4	13 (4.3%)
Necrotizing enterocolitis	10 (3.3%)
Parenteral nutrition (days)	11 (4–98)
Parenteral nutrition ≥14 days	88 (29.3%)
Intraventricular haemorrhage*	46 (15.3%)
Intraventricular haemorrhage grade 3 or 4*	12 (4.0%)
Cystic white matter disease*	3 (1.0%)
Cerebellar haemorrhage*	2 (0.7%)
Any finding neonatal ultrasound	50 (16.7%)
Postmenstrual age at discharge (weeks)	37.7 ±2.5

*diagnosed by neonatal ultrasound

### Frequency of brain injury

Of the total cohort of 300 infants 74 (24.7%) showed some form of brain injury on MRI at term-equivalent age. Of all infants 19 (6.3%) were diagnosed with any form of severe injury and 24 (8.0%) with more than one type of injury. Detailed results are presented in Table 2. The diagnosis IVH was already made by neonatal ultrasound in most cases (46 (95.8%) of 48 infants). In contrast, WMD and CBH were detected by neonatal ultrasound in only 8.0% to 10.0% of all cases (3 of 30 infants with WMD, 2 of 24 infants with CBH). WMD diagnosed by neonatal ultrasound was the cystic form in all three cases.

**Table 2:** Incidence of brain injury diagnosed by MRI at term equivalent age (n = 300).

Variable	n (%)
Any injury	74 (24.7%)
Any severe injury	19 (6.3%)
More than 1 injury type	24 (8.0%)
Intraventricular haemorrhage	48 (16.0%)
Severe intraventricular haemorrhage	5 (1.7%)
White matter disease	30 (10.0%)
Severe white matter disease	10 (3.3%)
Cerebellar haemorrhage	24 (8.0%)
Severe cerebellar haemorrhage	6 (2.0%)

### Injury patterns

**Fig 1:**
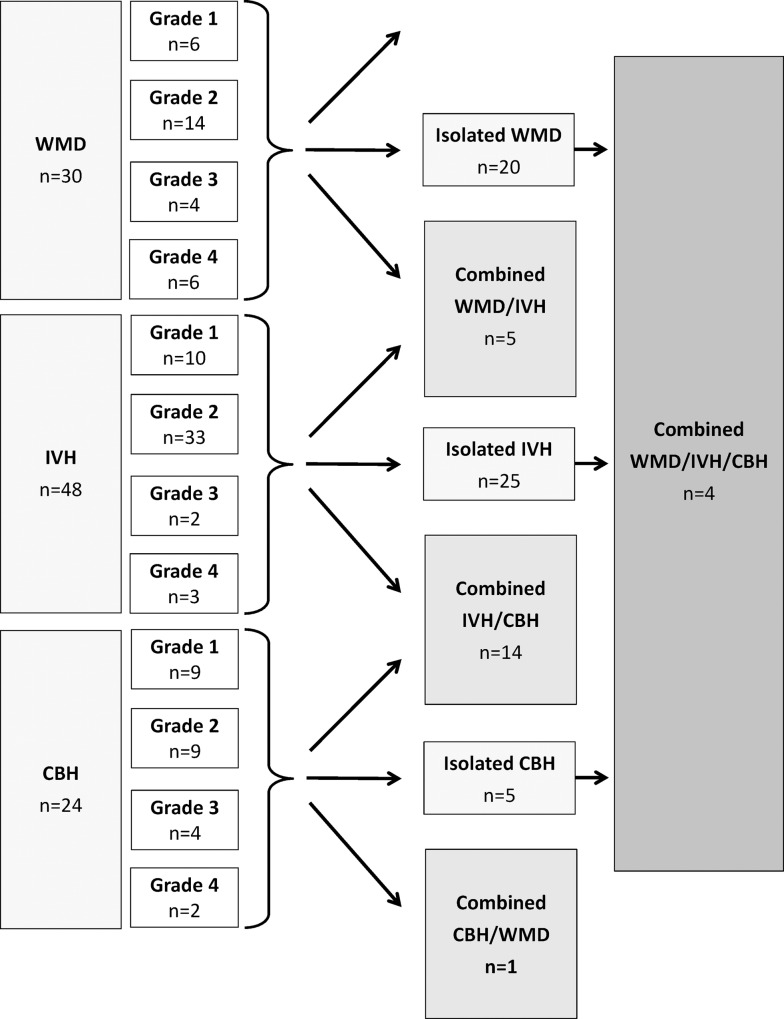
Patterns of brain injury diagnosed by MRI in very preterm infants at term equivalent age. Total n = 300, infants with injury n = 74.

IVH was the most frequent injury type observed (16.0% of all infants) and was an isolated finding in 62.5% of all cases. Similarly, WMD was an isolated finding in two-thirds (66.7%) of all cases. In contrast, CBH was frequently associated with an additional supratentorial injury (65.0%). Patterns of injury are visualised in Fig 1.

### Neonatal risk factors for brain injury

The rate of brain injury detected by MRI at term-equivalent age was higher in the group of more immature and sicker infants (Table 3).

**Table 3:** Neonatal risk factors for brain injury diagnosed by MRI at term equivalent age.

	No brain injury	MRI brain injury	p value
	(n = 226)	(n = 74)	
	n (%), mean (SD), median (range)		
Gestational age (weeks)	29.7 ±1.9	28.6 ±2.3	**<0.0001**
Gestational age <28 weeks	39 (13.0%)	21 (28.4%)	**0.045**
Birthweight (grams)	1287 ±387	1182 ±375	**0.042**
Small for gestational age	17 (7.5%)	5 (6.8%)	0.853
Birthweight <1000g	59 (26.1%)	23 (31.1%)	0.453
Male sex	112 (49.6%)	43 (58.1%)	0.229
Multiple birth	97 (42.9%)	32 (43.2%)	1.000
Caesarean delivery	213 (94.2%)	65(87.8%)	0.177
5’ Apgar <7	18 (8.0%)	14 (18.9%)	**0.030**
Antenatal steroids	211 (93.4%)	64 (86.5%)	0.056
Surfactant treatment	169 (74.8%)	64 (86.5%)	**0.037**
Ventilation (hours)	4 (0–506)	12 (0–1128)	**0.002**
Ventilation >6 hours	85 (37.6%)	50 (65.6%)	**<0.0001**
Nasal continuous positive airway pressure (days)	6 (0–71)	12 (1–72)	**0.012**
Need for supplemental O2 at day 28	50 (22.1%)	27 (35.5%)	**0.044**
Need for supplemental O2 at 36 weeks	16 (7.1%)	12 (16.2%)	0.056
Postnatal hydrocortisone treatment	23 (10.2%)	23 (31.1%)	**<0.0001**
Caffeine duration (postmenstrual age, weeks)	34.1 (32.0–43.9)	34.6 (32.4–40.4)	0.088
Preterm preterm rupture of membranes >24 hours	43 (19.0%)	11 (14.9%)	0.562
Early-onset sepsis	10 (4.4%)	5 (6.8%)	0.538
Late-onset sepsis	20 (8.8%)	11 (14.9%)	0.185
Patent ductus arteriosus	75 (33.2%)	27 (36.5%)	0.181
Patent ductus arteriosus surgical ligation	7 (3.1%)	4 (5.4%)	0.561
Catecholamine treatment	10 (4.4%)	15 (20.1%)	**<0.0001**
Retinopathy of prematurity grade 3 or 4	7 (3.1%)	6 (8.1%)	0.094
Necrotizing enterocolitis	7 (3.1%)	3 (4.1%)	0.712
Parenteral nutrition ≥14 days	59 (26.1%)	29 (39.2%)	0.062
Postmenstrual age at discharge (weeks)	37.4 ±2.2	38.7 ±3.1	**0.002**

Separate analysis of infants with severe injury showed similar associations and additionally revealed the need for supplemental oxygen at day 28 and at a postmenstrual age of 36 weeks, late-onset sepsis, patent ductus arteriosus, necrotising enterocolitis and parenteral nutrition for ≥14 days as neonatal risk factors for severe brain injury (data not shown).

We found that the prevalence of all common neonatal risk factors was greater within the group of infants with CBH than in infants without brain injury (Table 4). This pattern was not observed for infants with WMD. Mean gestational age did not differ significantly between infants with WMD and infants without brain injury (Table 4). The rate of infants with birthweight <1000g was 4-fold lower in infants with WMD. The only neonatal risk factor that was overrepresented within the group of infants with WMD was catecholamine treatment (p = 0.018).

**Table 4:** Neonatal risk factors for different types of brain injury diagnosed by MRI at term equivalent age.

	No brain injury	WMD		CBH		IVH	
	(n = 226)	(n = 30)	p value	(n = 24)	p value	(n = 48)	p value
Gestational age (weeks)	29.7 ±1.9	30.1 ±1.3	0.180	28.2 ±2.43	**<0.0001**	27.9 ±2.4	**<0.0001**
Gestational age <28 weeks	39 (13.0%)	2 (6.7%)	0.187	7 (29.2%)	0.167	18 (37.5%)	**0.003**
Birthweight (grams)	1287 ±387	1391 ±321	0.180	1088 ±354	**0.017**	1067 ±355	**<0.0001**
Small for gestational age	17 (7.5%)	0 (0.0%)	0.166	3 (12.5%)	0.621	5 (10.4%)	0.790
Birthweight <1000g	59 (26.1%)	2 (6.7%)	**0.021**	9 (37.5%)	0.236	20 (41.7%)	**0.036**
Male sex	112 (49.6%)	19 (63.3%)	0.177	16 (66.7%)	0.134	25 (52.1%)	0.874
Multiple birth	97 (42.9%)	15 (50.0%)	0.558	9 (37.5%)	0.669	16 (33.3%)	0.260
Caesarean delivery	213 (94.2%)	27 (90.0%)	0.555	19 (79.2%)	**0.013**	41 (85.4%)	0.086
5’ Apgar <7	18 (8.0%)	3 (10.0%)	0.766	8 (33.3%)	**<0.0001**	11 (22.9%)	**0.008**
Antenatal steroids	211 (93.4%)	28 (93.3%)	0.623	17 (70.8%)	**<0.0001**	41 (85.4%)	0.096
Surfactant treatment	169 (74.8%)	25 (83.3%)	0.370	20 (83.3%)	0.458	41 (85.4%)	0.134
Ventilation (hours)	4 (0–506)	8 (0–120)	0.149	13 (0–1128)	**0.018**	20 (0–1128)	**<0.0001**
Ventilation >6 hours	85 (37.6%)	16 (53.3%)	0.216	15 (62.5%)	0.056	33 (68.8%)	**<0.0001**
Nasal continuous positive airway pressure (days)	6 (0–71)	6 (1–27)	0.482	21 (3–70)	**0.002**	21 (1–72)	0.084
Need for supplemental O2 at day 28	50 (22.1%)	5 (16.7%)	0.734	12 (50.0%)	**0.011**	22 (45.8%)	**0.003**
Need for supplemental O2 at 36 weeks	16 (7.1%)	1 (3.3%)	0.690	7 (29.2%)	**0.002**	12 (25.0%)	**0.001**
Hydrocortisone for bronchopulmonary dysplasia	23 (10.2%)	4 (13.3%)	0.817	9 (37.5%)	**0.001**	20 (41.7%)	**<0.0001**
Caffeine duration (postmenstrual age, weeks)	34.1 (32.0–43.9)	34.3 (33.0–40.1)	0.972	34.9 (32.7–40.4)	**0.050**	34.9 (32.4–40.4)	**0.005**
Preterm preterm rupture of membranes >24 hours	43 (19.0%)	4 (13.3%)	0.473	4 (16.7%)	0.680	7 (14.6%)	0.739
Early-onset sepsis	10 (4.4%)	1 (3.3%)	1.000	0 (0.0%)	0.605	4 (8.3%)	0278
Late-onset sepsis	20 (8.8%)	1 (3.3%)	0.484	5 (20.8%)	0.075	9 (18.8%)	0.066
Patent ductus arteriosus	75 (33.2%)	7 (23.3%)	0.307	11 (45.8%)	0.259	19 (39.6%)	0.407
Patent ductus arteriosus surgical ligation	7 (3.1%)	0 (0.0%)	0.578	3 (12.5%)	0.060	3 (6.3%)	0.387
Catecholamine treatment	10 (4.4%)	5 (17.2%)	**0.018**	6 (26.1%)	**0.001**	11 (23.4%)	**<0.0001**
Retinopathy of prematurity °3–4	7 (3.1%)	1 (3.3%)	1.000	5 (20.8%)	**0.003**	6 (12.5%)	**0.014**
Necrotizing enterocolitis	7 (3.1%)	1 (3.3%)	1.000	2 (8.3%)	0.210	3 (6.3%)	0.387
Parenteral nutrition ≥14 days	59 (26.1%)	6 (20.0%)	0.568	12 (50.0%)	**0.022**	23 (47.9%)	**0.009**
Postmenstrual age at discharge (weeks)	37.4 ±2.2	37.9 ±2.6	0.406	40.1 ±3.6	**0.002**	38.9 ±3.4	**0.005**

IVH is usually detected by neonatal ultrasound and there is already an established set of neonatal risk factors for IVH. For completeness risk factors for IVH are listed in Table 4.

## Discussion

Our comprehensive assessment of a large and unselected cohort of very preterm infants proved that cerebral MRI at term-equivalent age, as addition to cranial ultrasound, detects brain injury in 25% of preterm survivors and especially facilitates the detection of WMD and CBH. MRI thereby contributes to specifying the type and frequency of injury patterns in this population. Analysis of neonatal characteristics revealed that the most immature infants with complicated neonatal history are at greatest risk for injury of the cerebellum. In contrast to this, infants with WMD turned out to not be part of this recognized group of most vulnerable infants. The only identifiable risk factor for this condition was the need for catecholamine treatment.

Several authors report good agreement between ultrasound and MRI regarding demonstration of IVH and cysts.[13] However, it has been shown that ultrasound lacks sensitivity in the detection of (punctate) cerebellar lesions and non-cystic WMD.[5, 14] This is in accordance with our own experience and confirmed by the results of the present study. The diagnosis of IVH was already made by ultrasound in most cases, whereas CBH were each diagnosed in only a fraction of all cases and non-cystic WMD was not detected by ultrasound at all.

Yet there is no other publication that used exactly Kidokoro’s definition of WMD. However, there are several reports that provide information on the incidence of punctate WM lesions that form the imaging correlate for Kidokoro’s WMD grades 1–3.[6, 14–17] The reported incidences range from 20% to 30%.[6, 14–17] There is some evidence that the total lesion burden of punctate WM lesions is better demonstrated in an early MRI scan (approx. three weeks after preterm birth) and there is a decrease in intensity and amount until term age.[6, 16, 18] Thus, in our study the performance of all MR scans during a narrow time window around 40 weeks postmenstrual age, thus 8 to 16 weeks after birth, might have led to underestimation of the total load of non-cystic WM injury.

Due to increased survival of highest-risk infants and better imaging modalities cerebellar injury is now reported more often, but established information about the frequency of CBH from either imaging modality in large cohort studies is still limited. Kidokoro found 10% of all infants to have a CBH and 2.2% a severe CBH.[8] This is in accordance with our own results. Using ultrasound, different working groups report an incidence that ranges up to 15%.[19] Studies using MRI report rates, depending on gestational age of the population included, as high as 20%, predominantly due to the detection of low-grade (punctate) lesions.[20] Furthermore, it has been shown that especially extremely immature infants are at high risk for developing concurrent IVH and CBH.[19] Also in our cohort we found additional supratentorial brain injury in about two-thirds of infants with CBH, with concurrent IVH appearing most frequently. This phenomenon may be explained by similarities in the pathogenesis of CBH and IVH, which are discussed below.

Speaking generally, the rate of brain injury was higher in the group of more immature infants with consequently higher rates of neonatal complications. Analysis of neonatal risk factors for the individual injury types revealed that CBH affected the most immature and sickest infants. Especially parameters for circulatory disturbance and respiratory disease were more common among infants with CBH. Similar observations have been made by other working groups that did extensive research on cerebellar injury in neonates. They proposed circulatory factors and severe respiratory problems to play a role in the onset of CBH.[19, 20] Whether these factors co-occur with CBH or whether and to what extent they are implicated in the pathogenesis of CBH has not yet been fully elucidated. However, it seems that the (multifactorial) pathogenesis of CBH in the preterm infant has similarities to that of IVH.[21] This assumption is also supported by our own analyses that revealed an overlapping of many clinical risk factors for CBH and IVH. Germinal matrices are present also in the cerebellum and analogous to the supratentorial germinal matrices these sites are especially vulnerable to circulatory disturbances, which are common in sick preterm neonates.[21]

Interestingly, this pattern of immaturity and related neonatal disease was not present in infants with WMD. Infants with WMD did not suffer more often from any neonatal disease with the exception of a higher rate of catecholamine treatment. The pathogenesis of non-cystic white matter injury is not yet completely understood, but the current evidence suggests both haemorrhagic and hypoxic-ischaemic processes to be involved in the pathogenesis of this lesion type.[17, 22] The role of circulatory factors and concurrent hypoxia-ischaemia is supported by a pathology study in infants with non-cystic WMD that showed diffuse gliosis suggestive of hypoxia-ischaemia.[23] This study was performed in term neonates with congenital heart disease, another group of infants in whom exactly this injury pattern is frequently found, and corroborates the hypothesis that altered cerebral perfusion may play a major role in the pathogenesis of non-cystic white matter injury. Need for catecholamine treatment may be regarded as a surrogate for severe neonatal diseases, e.g. arterial hypotension in the wake of sepsis. However, this assumption is reflected neither by our own data nor by the study by Kidokoro et al.[8] One reason may be the fact that there is a loss of efficient cerebral autoregulation during dopamine supply in preterm infants.[24] Additionally, it has been shown that preterm neonates treated for arterial hypotension with inotropic drugs, despite treatment, spent more time with a blood pressure below their gestational age than did age-matched controls that did not receive any blood pressure support.[25]

The main strength of this study is that the study cohort represents a consecutive, contemporary population seen at a well-equipped tertiary centre for neonatal care. A high percentage (84.5%) of the eligible population underwent MRI at term-equivalent age. Thus, the nature and frequency of cerebral findings may be regarded as representative for other European centres with comparable resources and concepts of care. Univariate analysis was chosen as an explorative approach to evaluate clinical risk factors for brain injury in our population. Adjustment for multiple testing was not considered to be reasonable since especially immaturity and parameters concerning respiratory disease are mutually dependent. A limitation at that point is that outcome data are not yet available for the total cohort. This data will be provided in the future since our cohort continues to be followed to school age and possibly beyond.

This study provides comprehensive data on frequency and patterns of brain abnormalities detected by conventional MRI at term-equivalent age in a contemporary cohort of preterm infants born at <32 weeks. There was good agreement between neonatal cranial ultrasound and MRI in the diagnosis of IVH, but MRI proved superior in the detection of CBH and non-cystic white matter injury. Decreasing gestational age and neonatal complications involved with immaturity have been identified as risk factors for CBH, whereas white matter injury was found in relatively mature infants and was associated with a more frequent need for catecholamine supply suggestive for circulatory disturbances.

Current evidence indicates an association between these early MRI findings and subsequent neurodevelopmental outcome. However, to date comprehensive assessment of the effect of especially isolated subtle brain injury and delayed maturation in otherwise “uncomplicated” and “healthy” preterm infants has been confined to the second year of life. Thus, after MRI at term-equivalent age has been implemented as routine examination in many centres, standardised neuropsychological follow-up of large cohorts of preterm infants until adulthood is absolutely essential to uncover potential associations between MRI findings and subtle cognitive deficits or behavioural problems that may first manifest themselves at later ages.
